# Habitat correlates of the Eurasian otter *Lutra lutra* recolonizing Central Poland

**DOI:** 10.1007/s13364-012-0107-8

**Published:** 2012-10-14

**Authors:** Jerzy Romanowski, Marcin Brzeziński, Michał Żmihorski

**Affiliations:** 1 Centre for Ecological Research, Polish Academy of Sciences, Dziekanów Leśny ul. Konopnickiej 1, 05-092 Łomianki, Poland; 2 Faculty of Biology and Environmental Studies UKSW, Wóycickiego 1/3, 01-938 Warsaw, Poland; 3 Department of Ecology, University of Warsaw, ul. Banacha 2, 02-097 Warsaw, Poland; 4 Museum and Institute of Zoology, Polish Academy of Sciences, ul. Wilcza 64, 00-679 Warsaw, Poland

**Keywords:** Recolonization, Habitat selection, Riparian zone, Indicator species

## Abstract

The increase in Eurasian otter *Lutra lutra* populations in their natural range and recolonization processes are recently observed in several European countries. We address the process of otter recolonization and habitat utilization in Central Poland over 14 years. Field surveys in 1998 and 2007 documented increase in occurrence of the species. The frequency of positive sites denoted 15 % in 1993, 38 % in 1998, and 89 % in 2007. Otter occurrence at study sites was positively affected by river width while negatively affected by presence of buildings at the site and river regulation. During the most intensive colonization process in the 1990s, the habitat preferences of the otter did not change. However, the sites inhabited by otters after 1998 were characterized by lower river width and tree cover and were more often located on regulated river sections, suggesting change in habitat tolerance during expansion. The otter abundance in transformed habitats is a result of increasing population numbers and the necessity to inhabit suboptimal sections of watercourses. Thus, it seems that presence–absence data for otter populations cannot be considered a reliable indicator of habitat quality, being depended of the population density.

## Introduction

The Eurasian otter *Lutra lutra* populations declined in large areas of Europe during the twentieth century (Macdonald and Mason 1988, 1994; Mason 1989; Lodé 1993; Kauhala 1996). However, the increase in the populations in their natural range (Sulkava et al. 2007) and recolonization processes are recently observed in several European countries (Green and Green 1997; Kranz et al. 2001; Mason and Macdonald 2004; Elmeros et al. 2006; McDonald et al. 2007; Prigioni et al. 2007). In the past, decline and/or extermination of otter populations was due to numerous reasons, including water pollution, PCBs concentrations, declining food resources, riparian habitat loss, hunting, poaching, road traffic, and fish trap mortality (Macdonald and Mason 1994; Mason 1989, 1997). The recent recovery of many otter populations is undoubtedly connected with reduction or elimination of at least some of these factors (Roos et al. 2001) and the conservation of the species (the Eurasian otter is partially protected in Poland). Thus, the habitat improvement and reduced otter mortality may have lead to the population growth, and as a consequence, to the enlargement of otter range in Europe. In Poland, where populations followed the same decline as in many other European countries, otters begun to increase in numbers and started to recolonize new areas in the 1980s (Włodek et al. 1989). In the first half of 1990s, as revealed by the first national survey, they were already widespread in most of the country; otter signs were recorded in 79.5 % of sites visited during the survey (Brzeziński et al. 1996). In that time, the only two large regions not inhabited by otters were the upper Odra River and Nysa Kłodzka River and their tributaries in south-west Poland and Bzura River catchment (excluding one tributary) in Central Poland. In 1990s, Bzura River was heavily polluted, and its many smaller tributaries were regulated and offered poor quality habitats to otters. On the other hand, the whole river catchment was surrounded by areas already inhabited by otters; thus, the background for natural recolonization process existed.

Eurasian otter abundance and distribution are related to a complex of environmental factors such as food availability, water quality, presence of riparian vegetation, human disturbance, etc. Habitats which sustain otter populations may be very diverse; however, all of them are characterized by the carrying capacity which enables foraging and breeding. Unfortunately, estimation of the quality of environmental parameters sufficient for existence of otter populations is difficult, the more so as riparian habitats often compose complicated system including more or less degraded patches. In general, unpolluted and unregulated rivers with well preserved riparian vegetation along the banks offering various shelters are considered to be optimal otter habitats. To what extend otters can use suboptimal habitats (i.e., significantly transformed by human activity) within the network of good quality habitats is still an open question. However, it seems obvious that population trends in the large-scale areas may significantly affect otter dispersal and movements and, thus, the tendency to enlarge their range. Therefore, identification of good quality habitats provides the framework to identify otter movement patterns within and between river basins and enables to characterize watercourses in terms of their ability to host otter source populations (Loy et al. 2009). The evidence-based knowledge concerning habitat requirements of the otter and habitat characteristics driving spatiotemporal variability of its population is crucial for effective conservation of the species. Moreover, bearing in mind potential otter–human interaction and conflicts in the context of fishery production (e.g., Sales-Luis et al. 2009; Kloskowski 2011), data on habitat correlates of the Eurasian otter are needed for proper population management of this species. The aim of this study was to examine the Eurasian otter population trends and to assess habitat correlates explaining occurrence of the species in Central Poland along two decades.

## Materials and methods

The study was undertaken in 7,800 km^2^ area of Bzura catchment in Central Poland. Bzura with the length of 166 km is one of the largest left tributaries of the River Vistula. The area is part of Masovian Plain, the low-lying region, relatively flat and with altitude ranging from 60 to 150 m asl (Kondracki 1988). Agriculture is the dominant land use in the area. The largest forested area is protected in Kampinos National Park located in the northeastern part of the study area. In its upper section, Bzura drains marshy valley with meadows and pastures. While most of the smaller tributaries are regulated, both Bzura and the largest tributary the River Rawka include seminatural sections with well preserved bank vegetation, which provide habitats for, e.g., waterfowl and beaver *Castor fiber*. Rivers in the catchment freeze in winter; the ice cover lasts for up to 60 days. The lowest levels of waters are observed in the July–August; the highest, in March–April (Kondracki 1988). Water level changes are pronounced; the discharge in the middle section of Bzura (near Sochaczew) varies from 2.5 to 480 m^3^/s (average 23). The average discharge of Bzura at efflux to Vistula is 28.6 m^3^/s. In the past, Bzura was heavily polluted by textile industry and communal waste waters and was considered one of the most polluted rivers in Poland. The water quality started to improve in the mid-1990s after construction of sewage treatment plants near main cities and industrial plants. The general improvement of water quality was confirmed by biological assessment using benthic diatoms in 2003, which demonstrated the recovery of species sensitive to organic water pollution (Szczepocka 2007). However, relatively high concentrations of nutrients, suspended matter, and heavy metals are still recorded in the river (WIOŚ 2007; Trawczyńska et al. 2009). A total of 32 species of fish were recorded in the catchment, including *Litophilus* species, e.g., *Cottus gobio* and *Phoxinus phoxinus* (Penczak 1968). Fast, natural regeneration of ichthyofauna in Bzura River started in 1990s. Recent studies revealed 22 fish species in Bzura River, including the most numerous *Rutilus rutilus*, *Perca fluviatilis*, and *Esox lucius* (Penczak et al. 2000).

Data on otter distribution were collected by visiting 212 sites in 1998 and 2007. Mean distance between nearest surveyed sites denoted 4.3 km (SD = 2.8 km). Among 212 sites studied, 49 were already surveyed by the same method and authors for otter presence during the national survey in 1993 (Brzeziński et al. 1996). The surveys were carried out from February till April each year, in the period of high detectability of otter signs (Romanowski 2000). The standard otter monitoring method was used to provide fully compatible results (Romanowski et al. 1996; Reuther et al. 2000). Searching usually started at a bridge. A maximum distance of 600 m was searched for the spraints and tracks of otters at the site. If no otter signs were found, the site was considered “negative.” At the majority of sites, as soon as otter signs were found, the search was stopped, and the site was coded as “positive.” In all cases, at least 200 m of river banks were surveyed to evaluate the characteristics of the site.

At each site, four environmental variables were recorded during the field observations in 1998 in order to describe habitat characteristics and identify factors that may have affected the occurrence of otters. The variables were evaluated for the whole 200–600-m section of surveyed bank and included the percentage of tree and shrub cover of the river bank (hereafter, *trees*), river width (*width*, log-transformed in the statistical analysis), river regulation (*regulation*, presence of embankment reinforcement), and presence of buildings (*buildings*).

**Fig. 1:**
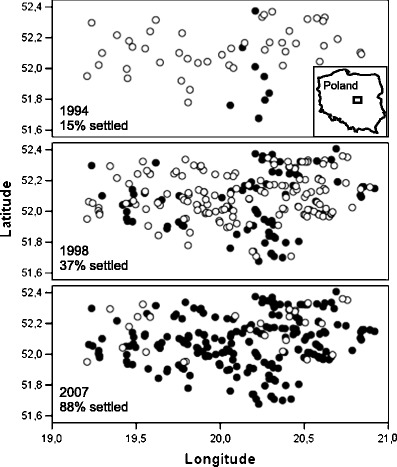
Distribution of sites surveyed for the presence of the Eurasian otter in Central Poland

In order to select habitat characteristics explaining occurrence of the otter on the basis of data from all 424 site visits (212 sites in 1998 and 2007; data from 1993 were excluded because of relatively small sample size), we implemented generalized linear models (GLM) with binomial error distribution and logit link. Moreover, as surveyed sites were placed relatively close to each other (Fig. 1), we corrected the modeling for possible spatial autocorrelation—we added spatial correlation structures to the model using Gaussian method for the parameterization for the correlation function. The modeling was implemented in R program (R Development Core Team 2010). We used presence/absence of otter as dependent binary variable and all four measured variables described above as explanatory variables, including both categorical factors and covariables. Additionally, year (*year*) was used as a fifth categorical independent variable. We made an attempt to check if the effects of particular habitat characteristic on the otter occurrence changed significantly between 1998 and 2007. For this purpose, we included in the GLM interaction terms between year and four remaining predictors. We removed insignificant interaction terms from the final model.

Finally, we compared habitat characteristics of “presence” sites (settled by the otter in 1998; *n* = 79) with “colonization” sites (sites where the otter was not recorded in 1998 and was recorded in 2007; *n* = 110). For the comparison, permutation test of equality and *χ* ^2^ test were used, and kernel density method, for the visualization (R Development Core Team 2010).

## Results

The otter has expanded its distribution range across Central Poland in the last two decades (Fig. 1). The frequency of positive sites denoted 15 % in 1993, 37 % in 1998, and 88 % in 2007, and the difference was highly significant (Chi-square test, *χ* ^2^ = 163.17; degrees of freedom (*df*) = 2; *p* < 0.0001). For the 49 sites surveyed in the three periods (i.e., 1993, 1998, and 2007), the difference in the occurrence of the Eurasian otter was also highly significant (*χ* ^2^ = 25.80; *df* = 2; *p* < 0.0001).

**Fig. 2:**
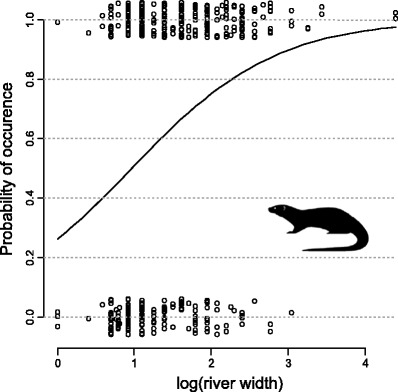
The effect of river width (log-transformed) on the probability of the Eurasian otter occurrence. Random noise was added along the *y*-axis to avoid point overplotting

Interaction between year and all remaining predictors (trees, width, regulation, and buildings) was insignificant (*p* > 0.2 in all cases) and was removed from the final model. In the GLM, the year of the study significantly explained the probability of the otter occurrence in the studied sites (Table 1). Besides strong effect of the year, also river width significantly positively affected the probability of occurrence of the otter (Table 1, Fig. 2). In contrary, presence of buildings and river regulation affected negatively the probability of occurrence (Table 1). Percentage of tree and shrub cover of the river bank seems to have no effect.

**Table 1:** Parameter estimates (odds ratio and their 95 % confidence intervals) and their significance of the generalized linear model explaining occurrence of the Eurasian otter in the studied sites in Central Poland

Source	Exp (B)	95 % CI	*P*
Intercept	0.101	0.042; 0.246	<0.0001
Width	4.216	2.759; 6.443	<0.0001
Trees	1.013	0.996; 1.030	0.1272
Year			
2007	20.409	11.347; 36.708	<0.0001
1998	1		
Regulation			
Yes	0.528	0.262; 1.063	0.0744
No	1		
Buildings			
Yes	0.515	0.269; 0.984	0.0453
No	1

**Fig. 3:**
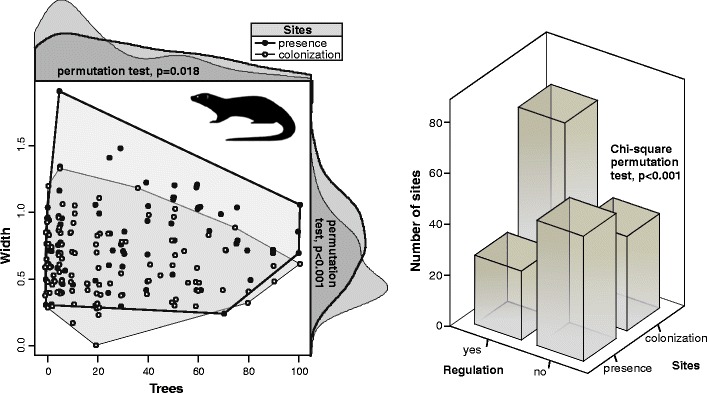
Habitat characteristics of Eurasian otter sites in Central Poland. Result of the permutation tests comparing habitat features of presence and colonization sites is given on the plot

Comparison of presence and colonization sites shows that the later was characterized by a lower river width and lower percentage of tree cover (Fig. 3). Among sites surveyed, 67 % of river banks at presence sites were not regulated, whereas analogical value for the colonization sites denoted 33 %, and the difference was highly significant (Chi-square permutation test, *p* < 0.001; Fig. 3). Buildings were present in 16 % of the presence sites and in 22 % of colonization sites, but the difference was not significant (Chi-square permutation test, *p* = 0.468).

## Discussion

Our study revealed the rapid increase of the Eurasian otter in the Bzura catchment in Central Poland. Percentage of sites where otter signs were recorded increased over fivefold during 14 years. The increase of the otter population in Poland was already recorded at the beginning of 1990s during the national survey (Brzeziński et al. 1996); however, in that period, some regions of the country, including Bzura catchment, were still uninhabited by otters, or their abundance was extremely low.

Occurrence of the otter in the study area was significantly explained by several habitat characteristics. The species preferred wide, afforested watercourses, whereas regulation and presence of buildings negatively affected its occurrence. The results stay in accordance to former investigations in this issue, including earlier data from Central and Eastern Poland (Romanowski 2006).

In general, increase of riparian habitat quality is thought to be one of the most important drivers of the large-scaled restoration of the species (Elmeros et al. 2006; Clavero et al. 2010). In West European countries, reintroduction programs in the areas where otters have been exterminated are developed after restoration of natural otter habitats and improving water quality (Koelewijn et al. 2010). Several other efforts, such as modification of fyke nets and creating fauna passages, may also contribute to the recovery of otter populations (Elmeros et al. 2006).

In contrary to former research, our findings seem to indicate that changes in the habitat quality did not contribute in such extant to the rapid recolonization of the River Bzura by the otter. The spread was rather driven by the overall increase in species range and numbers in Poland (Romanowski 2006), simultaneous to the process in many other European countries (Elmeros et al. 2006; Sulkava et al. 2007; Clavero et al. 2010). According to our observations during consecutive field surveys, no significant positive changes in riparian habitats in Bzura catchment were observed since 1990s. River Bzura remains the most heavily polluted river in Central Poland, and it tributaries are under heavy pressure from agricultural and council origin sewage (WIOŚ 2007). For example, in Sochaczew Province, where many sites became inhabited by the otter after 1998, sewage production of industrial origin which was channeled directly to aquatic systems increased from 580,000 to 1,263,000 m^3^/year in the period from 1998 to 2007 (CSO 2008). Also, production of sewages of council origin increased nearly threefold during this period—from 10,930 kg of N/year in 1998 to 30,609 kg of N/year in 2007 (CSO 2008). Despite habitat quality did not improve distinctly in the study area, the results show that otters were expanding their range in Central Poland, and signs of their presence were observed in 110 new localities. Therefore, we assume that the increase in abundance of the otter is unlikely to be explained by the large-scaled habitat transformations, and the recolonization was driven mainly by the habitat plasticity of the species and ability to survive in suboptimal habitats.

Comparison of sites inhabited in 1998, and those inhabited in 2007 showed that the Eurasian otter extended significantly its habitat tolerance. At the beginning of recolonization process in the period from 1994 to 1998, the individuals selected habitats of highest quality. During that time, otters were recorded at presence sites located predominantly along wide and unregulated watercourses. However, following the increase in occurrence and range during later phase of recolonization, otters were more frequently detected in low-quality habitats (e.g., regulated river sections). As a result, characteristics of sites inhabited by the otter in 1998 and colonized in the period from 1998 to 2007 differed greatly. Similar observations were reported in Scotland, where increasing otter population recolonized relatively polluted and industrialized area (Green and Green 1997). Wide distribution of otter signs in various types of riparian habitats, including these strongly transformed by human activity, may be an indicator of thriving and numerous population (Baltrūnaitė et al. 2009). The otter abundance in poor-quality habitats is a result of increasing population numbers and the necessity to inhabit suboptimal sections of watercourses. Such ecological mechanism seems to be typical for each population of growing density, which previously inhabited only optimal patches of heterogeneous habitat (Fretwell 1972). Indeed, such differences in habitat selection were documented for the two areas with low and high otter abundance; in low-abundance area (Central Poland), otters preferred unregulated rivers with vegetated banks, located in proximity to forest and fish ponds, while in high abundance area (Eastern Poland), otters occupied all types of aquatic habitats according to their availability (Romanowski 2006). Frequent use of human-transformed habitats, e.g., regulated rivers, reclamation ditches, and artificial reservoirs, was documented in most recent survey in Lithuania, most probably reflecting high otter numbers in the country (Baltrūnaitė et al. 2009). However, increased utilization of suboptimal patches of habitat is more probable if they are incorporated within the network of the well-preserved habitats.

Carrying capacity of otter habitats in a large distribution range may differ in particular areas, and the high-quality habitat patches are usually sites of source populations for the surrounding areas of lower quality (Sulkava et al. 2007). The recolonized Bzura catchment offers lower-quality habitats as compared to many other Polish rivers. However, some sections of the main river and several tributaries have not been subjected to serious damage and survived in relatively good condition. Moreover, Bzura, on its own, is a tributary of the largest Polish river—Vistula—which is inhabited by a thriving otter population (Brzeziński et al. 1996). Thus, sink populations (sensu Pulliam 1988) in Bzura catchment may be constantly supplied by otters dispersing from good-quality habitats.

In general, otter densities are positive, while their home-range sizes are inversely related to the river width (Sidorovich et al. 1996; Ó Néill et al. 2009), which is mainly a consequence of the higher fish productivity in larger rivers. On very small watercourses, otters are usually occasional visitors. Very small rivers can be used as feeding grounds or migratory routes; however, they cannot sustain sedentary breeding population. Sulkava et al. (2007) reported that small river systems were inhabited by otters temporary and abandoned during winter, and that probability of successful breeding in the suboptimal habitats was very low. Summarizing, poor-quality habitats are being visited occasionally by otters, and thus, environmental analysis which attempt to explain otter distribution based on the abundance of otter signs may give a large proportion of unexplained variation (Jenkins and Burrows 1980; Macdonald and Mason 1983).

The Eurasian otter was considered as a flagship species and indicator of high-quality aquatic habitats (Macdonald and Mason 1994; Cianfrani et al. 2011). Several studies confirmed that the otters avoid highly polluted water bodies and, during decades, had persisted in the landscape of low anthropogenic impact. Recent studies indicate that the recovery of the species is accompanied by change in habitat selection (Romanowski 2006; Clavero et al. 2010). Baltrūnaitė et al. (2009) reported that, in Lithuania, where otters are widely distributed, the presence of human settlements negatively affected the otter abundance, but the regulation of watercourses did not. Actually, our results partially support the statements since we recorded that the species was less frequent at river sections with high human activity and embankment reinforcements.

Madsen and Prang (2001) and Delibes et al. (2009) concluded that otter must be treated with care as an indicator of good aquatic habitat quality. Despite that our research did not consider several important environmental parameters, such as water quality or food supply, the evident change in tolerance to habitat characteristics recorded in our study leads to a similar conclusion that presence–absence data for expanding otter populations should not be considered as a reliable indicator of habitat quality.
